# In-Depth Transcriptome Sequencing of Mexican Lime Trees Infected with *Candidatus* Phytoplasma aurantifolia

**DOI:** 10.1371/journal.pone.0130425

**Published:** 2015-07-01

**Authors:** Mohsen Mardi, Laleh Karimi Farsad, Javad Gharechahi, Ghasem Hosseini Salekdeh

**Affiliations:** 1 Department of Systems Biology, Agricultural Biotechnology Research Institute of Iran, Karaj, Tehran, Iran; 2 Chemical Injuries Research Center, Baqiyatallah University of Medical Sciences, Tehran, Iran; 3 Department of Molecular Systems Biology at Cell Science Research Center, Royan Institute for Stem Cell Biology and Technology, ACECR, Tehran, Iran; Academia Sinica, TAIWAN

## Abstract

Witches’ broom disease of acid lime greatly affects the production of Mexican lime in Iran. It is caused by a phytoplasma (*Candidatus* Phytoplasma aurantifolia). However, the molecular mechanisms that underlie phytoplasma pathogenicity and the mode of interactions with host plants are largely unknown. Here, high-throughput transcriptome sequencing was conducted to explore gene expression signatures associated with phytoplasma infection in Mexican lime trees. We assembled 78,185 unique transcript sequences (unigenes) with an average length of 530 nt. Of these, 41,805 (53.4%) were annotated against the NCBI non-redundant (nr) protein database using a BLASTx search (e-value ≤ 1e-5). When the abundances of unigenes in healthy and infected plants were compared, 2,805 transcripts showed significant differences (false discovery rate ≤ 0.001 and log_2_ ratio ≥ 1.5). These differentially expressed genes (DEGs) were significantly enriched in 43 KEGG metabolic and regulatory pathways. The up-regulated DEGs were mainly categorized into pathways with possible implication in plant-pathogen interaction, including cell wall biogenesis and degradation, sucrose metabolism, secondary metabolism, hormone biosynthesis and signalling, amino acid and lipid metabolism, while down-regulated DEGs were predominantly enriched in ubiquitin proteolysis and oxidative phosphorylation pathways. Our analysis provides novel insight into the molecular pathways that are deregulated during the host-pathogen interaction in Mexican lime trees infected by phytoplasma. The findings can be valuable for unravelling the molecular mechanisms of plant-phytoplasma interactions and can pave the way for engineering lime trees with resistance to witches’ broom disease.

## Introduction

Mexican lime (*Citrus aurantifolia* L.) is one of the most economically important citrus trees grown in the south of Iran. The production of its fruit has been markedly reduced by a severe epidemic of the devastating witches’ broom disease of lime (WBDL) in Iran and adjoining countries [1]. This disease was first reported in Oman during 1980s [2] and then spread to other countries where lime is cultivated, such as Iran [3], United Arab Emirates [4], Saudi Arabia [5], India [6], and Pakistan [7]. It is estimated that over 98% of lime cultivation is affected by this disease [8]. Affected trees usually develop secondary shoots (so-called witches’ brooms) with short internodes and many small pale-green to yellow leaves. In the advanced stages of this disease, all shoots develop witches’ brooms and the tree eventually declines within four to five years [9]. A candidatus phytoplasma (*Candidatus* phytoplasma aurantifolia) has been identified as the causative agent of WBDL [10]. It is an obligate biotrophic mycoplasma-like organism that completely depends on living host cells for its nutritional requirements. Phytoplasmas have a minimal genome that lacks many essential genes that encode for components of metabolic pathways; they are thus unlikely to be able to synthesise nucleotides, amino acids, and fatty acids, so these must be imported from the host plant [11]. Phytoplasmas are the only known organisms that lack ATP-synthase subunits, which are thought to be essential for life [11]. However, there are major obstacles to the detailed characterisation of phytoplasmas and identification of the molecular mechanisms behind their pathogenicity and mode of interaction with host plants owing to the inability to culture them *in vitro* and their inaccessibility in host plants [11,12].

The advent of high-throughput ‘omics’ technologies for the detection, quantification, and identification of biological molecules has provided novel opportunities for the discovery of genes and molecular mechanisms that are involved in plant-pathogen interactions. In recent years, our group has applied several omics-based approaches to study the molecular responses of Mexican lime trees to *Ca*. P. aurantifolia infection. In one study, Zamharir *et al*. [13] applied a cDNA-amplified fragment length polymorphism (cDNA-AFLP) to study gene transcripts in Mexican lime trees that are differentially expressed during infection by *Ca*. P. aurantifolia. In addition, two-dimensional gel electrophoresis and quantitative label-free-based proteomics were also applied to investigate the proteomic changes associated with *Ca*. P. aurantifolia infection [14,15]. Although these studies have provided a snapshot of gene expression and regulation during infection of Mexican lime with *Ca*. P. aurantifolia, the scale of these studies was generally limited and they could not define the key regulatory genes that play an important role in the interaction of phytoplasma with its host plants.

The emergence of high-throughput next-generation sequencing (NGS) technologies has revolutionised genomic research in recent years. The current NGS platforms have allowed in-depth transcriptome sequencing of nearly all plant species, even those with complex genomes (such as polyploids), those without genomic or EST sequences, and those in which genome sequencing is not cost-efficient [16]. Interestingly, these approaches have allowed to generate millions of short cDNA reads that can be assembled to recover full-length genes, novel transcripts, splicing variants, and expressed single nucleotide polymorphisms (SNP) in different plant tissues or under various stress conditions [17–19]. In addition, transcriptome sequencing enables absolute measurement of gene expression, which, compared to relative quantification using microarrays, offers more useful data and greater accuracy [19–21]. In recent studies, transcriptome sequencing has been applied to explore molecular pathways affected during plant-pathogen interactions in plant species such as citrus [22], *Arabidopsis* [23], potato [24], cotton [25], *Nicotiana tabacum* [26], and *Paulownia* [27,28].

The aims of this study were to generate detailed transcriptome sequences that can be used in future genomic and transcriptomic studies of Mexican lime and to identify genes that are differentially expressed during phytoplasma infection. By comparison of transcript levels of healthy and infected Mexican lime trees, we identified 2,805 transcripts whose expression was deregulated due to infection by *Ca*. P. aurantifolia. We thus provide evidence on the mechanisms behind the interaction of Mexican lime with *Ca*. P. aurantifolia.

## Material and Methods

### Plant material

Mexican lime seedlings were grown in an insect-free greenhouse at a temperature of 25–28°C and relative humidity of 50%. The phytoplasma strain that was used for disease development had first been detected in a field grown tree about six years ago in an orchard of Mexican lime in Bandar Abbas, Hormozgan province. The strain was maintained on Mexican lime seedlings growing under controlled greenhouse conditions. To generate infected plants, bud sticks from Mexican lime trees infected with *Ca*. P. aurantifolia were grafted on healthy trees. To minimize the effect of grafting on our transcriptome analysis, bud sticks from healthy trees that had never been infected with *Ca*. P. aurantifolia were grafted on healthy trees and the grafted plants were used as a control (disease-free plants). The grafted plants were covered with plastic bags for one month to increase the relative humidity and to promote the growth of grafted buds. Mexican lime trees that had been grafted with at least five infected specimens developed typical symptoms of witches’ broom disease within less than five months. Approximately 20 weeks after grafting, leaf samples from those infected plants that showed typical symptoms of WBDL and those control plants without any disease symptoms were harvested. Leaf samples from five diseased and five healthy plants were pooled separately, snap-frozen in liquid nitrogen and stored at –80°C until RNA extraction.

### Molecular detection of phytoplasma infection by nested PCR

The presence/absence of the phytoplasma in the inoculated and control plants was confirmed using 16S rRNA gene amplification and sequence analysis. To this end, total DNA was extracted from leaf samples as described previously [13]. A region within the phytoplasma 16S rRNA gene was PCR amplified using primers P1 (5’-AAGAGTTTGATCCTGGCTCAGGATT-3’) [29] and P7 (5’-CGTCCTTCATCGGCTCTT-3’) [30] and the resulting P1–P7 amplicon was diluted (1/100) and used as template DNA for nested PCR amplification using the universal primer pair for phytoplasmas r16r2/r16F2n [31]. The resulting amplicon was then sequenced using the big dye labelling method. The 16S rRNA sequences were then homologue searched against 16S rRNA gene sequence databases.

### RNA extraction, cDNA library preparation, and sequencing

Total RNA from five healthy and five infected leaf samples was extracted using the RNeasy plant mini kit (Qiagen, CA, USA). Genomic DNA contamination was eliminated using RNase-free DNase I (Qiagen) treatment. RNA quantity and quality were determined using a NanoDrop ND 1000 spectrophotometer (Thermo Scientific, MA, USA) and agarose gel electrophoresis, respectively. For each library (healthy and infected) 20 μg of total RNA from five replicate samples were equally pooled and the resulting pooled RNAs were used for cDNA library preparation. The cDNA library construction and sequencing were performed at the Beijing Genome Institute (BGI), Shenzhen, China. Briefly, poly(A) mRNAs were enriched from 20 μg of total RNA using biotin-oligo(dT) magnetic bead adsorption. To avoid priming bias, the purified mRNA was first fragmented into 200–700-nt fragments by incubation with divalent cations at 94°C for 5 min. Then, the double-stranded cDNA was synthesised by priming with random hexamer primers using the Super Script Double-Stranded cDNA Synthesis kit (Invitrogen, CA, USA), purified with a QiaQuick PCR extraction kit (Qiagen), and then washed with EB buffer for end repair and single-nucleotide adenine addition. The synthesised cDNA was subjected to end repair and phosphorylation using T4 DNA polymerase, Klenow DNA polymerase, and T4 polynucleotide kinase. The repaired cDNA fragments were then 3’-adenylated using Klenow Exo- (3’ to 5’ exo minus, Illumina). Finally, Illumina paired-end adapters were ligated to the ends of these 3’-adenylated cDNA fragments, and the required fragments were purified by agarose gel electrophoresis and enriched by PCR amplification to construct the cDNA library. Finally, the library was loaded onto the channels of an Illumina HiSeq 2000 instrument, and the sequencing-derived raw image data were transformed by base calling into sequence data using Illumina pipeline software v1.6. Each paired-end library had an insert size of 200–700 bp. The average read length of 90 nt was generated as raw data. The data sets are available at the NCBI SRA database under the accession number: SRA058604. Clean reads were obtained from raw data by filtering out adapter-only reads, reads with unknown sequences ‘N’, and reads containing more than 50% of bases with a q-value ≤20. Transcriptome *de novo* assembly was performed using the SOAP *de novo* software [32]. The resulting contigs were then further assembled into scaffolds using CAP3 [33]. After gap filling using paired-end information, the scaffolds were assembled into 78,185 unigenes. Sequence directions of the resulting unigenes were determined by performing BLASTx (e-value <1e-5) searches against protein databases, with the priority order of nr (non-redundant protein sequences at the NCBI), Swiss-Prot, Kyoto Encyclopedia of Genes and Genomes (KEGG), and the database of Clusters of Orthologous Groups of proteins (COG) (e-value ≤1e-5), if conflicting results were obtained. When a unigene happened not to be aligned to any of the above databases, ESTScan software was used to predict its coding regions, as well as to determine its sequence direction [34].

### Gene annotation and functional classification

To assign molecular functions to transcripts sequences, the nucleotide sequences of unigenes were blast-searched against the NCBI nr, Swiss-Prot, and KEGG databases using the BLASTx algorithm. The significant e-value cut-off was set as ≤1e-5. The resulting BLASTx hits from the nr database were processed using the Blast2GO software [35] to retrieve functional annotations by gene ontology (GO) terms describing higher-level GO terms of molecular function, biological process, and cellular component ontologies. The WEGO software was used to perform GO functional classification for unigenes that matched to GO terms and to understand the distribution of gene functions at the macro level [36]. Domain-based alignments were performed against the COG database at the NCBI with an e-value cut-off of 1e-5. The KEGG pathway annotation was also performed by sequence comparisons against the KEGG database using the BLASTx algorithm with the same e-value cut-off [37].

For gene expression analysis, the transcript abundances of all assembled unigenes in the two libraries (healthy (H) and infected (I)) were normalised using the RPKM (the number of reads per kilobase of exon region per million mapped reads) method, which provides an estimate of expression level by taking into account variation in transcript length [19]. The probability of a transcript being expressed equally between H and I libraries was calculated as described previously [38] using the following equation:

$$p\left(n|m\right)={\left(\frac{N_{I}}{N_{H}}\right)}^{n}\frac{\left(m+n\right)!}{m!n!{\left(1+\frac{N_{I}}{N_{H}}\right)}^{m+n+1}}$$

Where the calculated *p*-value corresponds to the statistical significance level of differential expression, *N*_*I*_ and *N*_*H*_ refer to the total number of clean reads in I and H libraries, respectively, and n and m represent the total number of reads mapped in I and H libraries, respectively. The false discovery rate (FDR) was used to determine the *p*-value threshold. The ratio of I-RPKM to H-RPKM for each transcript was log2 transformed to identify candidate differentially expressed genes (DEGs). We applied stringent statistical criteria for the identification of candidate DEGs. Only those transcripts that showed statistically significant differences with an FDR ≤0.001 and a log2 ratio ≥1.5 were accepted as candidate DEGs. Gene expression patterns were visualised using the MAPMAN software [39]. In addition, KEGG pathway analysis was performed to map differentially expressed genes to biological pathways. Pathway analysis can improve our understanding of molecular events in plants by providing integrative information about the coordinated action of differentially expressed genes in a particular cellular context.

### Quantitative real-time PCR (qRT-PCR) validation of the expression of a selected set of DEGs

Total RNA was extracted from three biological replicates of infected and healthy Mexican lime trees as described above. One microgram of total RNA from each replicate was treated with 1 unit of DNase I (Qiagen) and reverse-transcribed in a 20-μl reaction using the iScript cDNA Synthesis Kit (Bio-Rad, CA, USA) in accordance with the manufacturer’s instructions. Gene-specific primers were designed for 25 genes that mostly belonged to key pathways with possible implication in disease progression and resistance. The sequence of primers along with the length of amplicon and annealing temperature for each primer pairs are presented in Table A in S1 File. The selected genes were quantified using the MyiQ Single-Color Real-Time PCR Detection System (Bio-Rad) with a single peak on the melting curve to ensure a single product. At least three biological and three technical replicates were run from infected and healthy samples. qRT-PCR was performed in a 25-μl reaction containing 12.5 μl of Power SYBR Green PCR Master Mix, 1 μl of each primer (10 mM), 3 μl of template cDNA (1/50 dilution), and 8.5 μl of dH_2_O. The thermal cycling conditions consisted of an initial denaturation at 95°C for 5 min, followed by 40 cycles of denaturation at 95°C for 30 s, annealing at 59–62°C for 30 s, and extension at 72°C for 45 s, and a final extension step at 72°C for 5 min. The change in the expression of selected transcripts was measured using the comparative cycle threshold method with the cycle threshold value of the internal control gene for each sample as a standard [40]. The 18S rRNA gene was used as an internal housekeeping gene for data normalisation.

## Results and Discussion

### Characterization of phytoplasma infected plants

**Fig 1:**
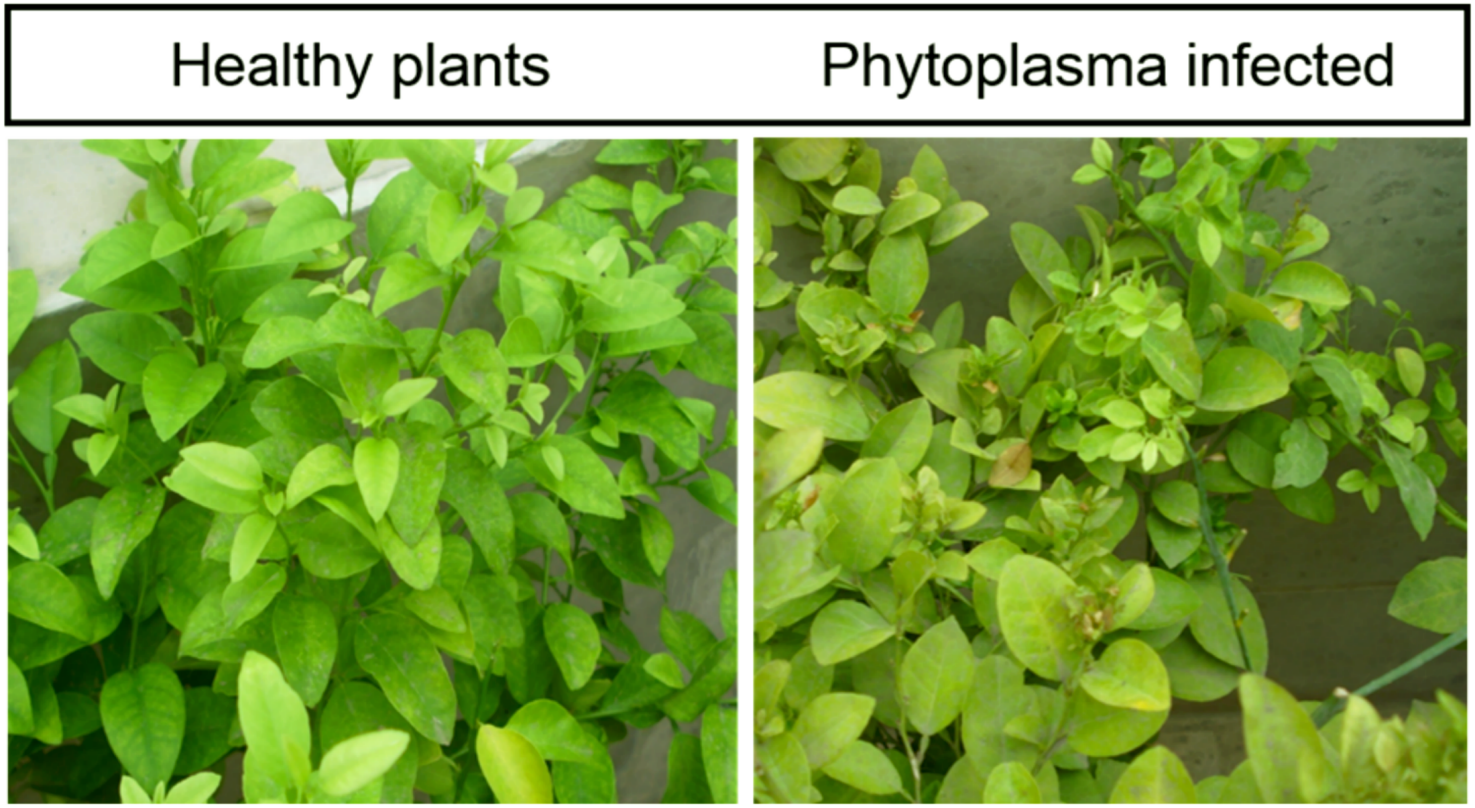
The overall phenotype of healthy and phytoplasma-infected plants. Diseased plants developed typical symptoms of witches’ broom disease, including the development of secondary shoots with small internodes covered by many small pale-green to yellow leaves and exhibiting a yellowish appearance over their entire surface. Disease symptoms developed within five months after grafting.

Healthy seedlings that had been grafted with infected bud sticks showed typical symptoms of witches’ broom disease, including the development of small secondary shoots with short internodes and many small yellow and pale-green leaves, overall yellowing of leaves, and sterile flowers, within five months after grafting (Fig 1). Disease-free plants (healthy) are also shown for comparison. PCR amplification and sequencing of the phytoplasma 16S rRNA gene from DNA samples of infected seedlings confirmed the presence of *Ca*. P. aurantifolia. Sequence similarity analysis of the 16S rRNA gene amplicon using iPhyClassifier software showed that it was generally similar to the reference 16Sr group II, subgroup B phytoplasma (GenBank accession: U15442), with a coefficient of pattern similarity of 0.99. This result confirmed that the 16S rRNA gene sequence detected in the infected plants was amplified from a variant of 16SrII-B, which was mostly related to *Ca*. P. aurantifolia.

### Transcriptome sequencing and assembly

To identify candidate genes that are differentially expressed in Mexican lime trees infected with *Ca*. P. aurantifolia 2×54,177,778 paired-end reads with a length of 90 nt were generated using an Illimina Hiseq2000. After filtering for low-quality reads, totals of 17,242,556 and 15,364,416 clean reads were kept for infected and healthy libraries, respectively. After gap filling using paired-end information, a total of 78,185 unique transcript sequences (unigenes) with an average length of 530 nt were detected in the assembled scaffolds (Table B in S1 File). Length distribution analysis showed that more than 40% of the unigenes were between 200 and 300 nt in length (Figure A in S2 File). In addition, more than 84% (66,076) of unigenes showed no gaps (Figure A in S2 File).

### Functional annotation using homology search

**Fig 2:**
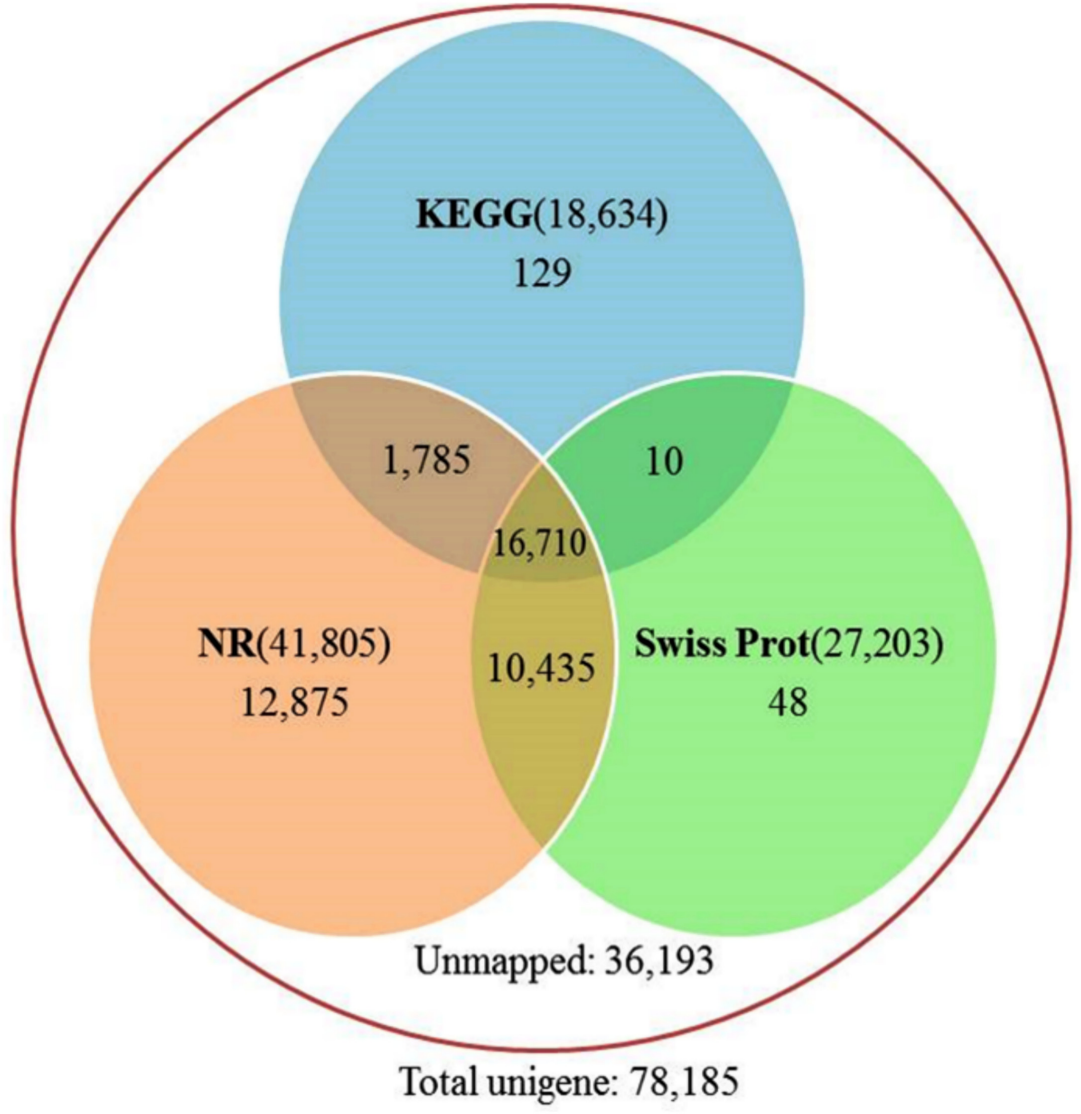
Venn diagram showing the total number of annotated unigenes in each probed database (circles). The number within parentheses in the non-overlapping region of each circle shows the total number of annotated unigenes in that database and the number outside of parentheses shows the total unigenes that uniquely matched with hits from that database. The number in overlapping regions shows the total number of unigenes that were simultaneously annotated in two or three databases.

For functional annotation, the nucleotide sequences of assembled unigenes were searched against the NCBI nr, Swiss-Prot, and KEGG databases using the BLASTx algorithm (e-value ≤1e-5). As a result, 41,805 (53.4%), 27,203 (34.8%), and 18,634 (23.8%) unigenes showed significant matches to known proteins in the nr, Swiss-prot, and KEGG, respectively (Fig 2). Only 21% of unigenes (16,710) were annotated by all three databases, 13% (10,435) by nr and Swiss-Prot, 2% (1,785) by nr and KEGG, and 0.01% (10) by Swiss-Prot and KEGG databases (Fig 2). A total of 36,193 unigenes (46%) did not show any significant matches to known proteins in these databases and presumably represent species-specific transcripts that did not have any homologue in the database or may be derived from non-conserved or untranslated regions of genes. However, the sequence length of unigenes may also affect the blast search output, since a large fraction of unannotated unigenes (~90%) were found to be around 200–500 bp in length. Analysis of species origin of top hits showed that more than 80% were matched with significantly low e-values to sequences from eight species with well-annotated genomes (*Arabidopsis thaliana* (44%), *Oryza sativa* (11%), *Populus trichocarpa* (11%), *Vitis vinifera* (5%), *Medicago truncatula* (3%), *Ricinus communis* (2%), *Citrus* (2%), and *Glycine max* (2%)) (Figure B in S2 File). This further highlights the robustness and reliability of our annotation process.

A total of 18,336 unigenes (23%) were assigned to at least one GO term and were classified into different functional categories using the WEGO software [36]. On the basis of the sequence similarity with top hits from the nr database, GO-assigned unigenes were classified into three high-level GO categories: biological process, molecular function, and cellular component (Figure C in S2 File). A total of 11,736 unigenes with BLAST hits were further assigned to 25 functional categories based on the COG classification (Figure D in S2 File).

### Identification and functional characterization of differentially expressed genes (DEGs)

When the expression levels of unigenes were compared in healthy and infected libraries, a total of 2,805 unigenes showed significant differences in abundance (FDR ≤0.001 and log_2_ ratio ≥1.5). Of these, 1,943 were up-regulated and 862 were down-regulated in response to phytoplasma infection. Furthermore, 199 and 94 unique transcripts were detected exclusively in the healthy or infected plants, respectively. Interestingly, more than 83% (2,334) and 43% (1,209) of DEGs were matched to at least one hit in the NCBI nr and Swiss-Prot databases, respectively. KEGG pathway analysis showed that a total of 939 DEGs were enriched in 43 metabolic and regulatory pathways (q-value ≤0.05) (Table 1). The pathways that are mostly enriched with upregulated DEGs include metabolic pathway (378), biosynthesis of secondary metabolites (237), plant-pathogen interaction (118), starch and sucrose metabolism (92), amino acid metabolism (87), plant hormone signal transduction (34), and fatty acid metabolism (15). The pathway for ubiquitin mediated proteolysis (7) and oxidative phosphorylation (16) are significantly enriched in down-regulated unigenes.

**Table 1:** Differentially expressed unigenes were enriched in 43 KEGG metabolic and regulatory pathways.

Pathway	KEGG ID	Number of up-regulated unigenes	Number of down-regulated unigenes
Metabolic pathways	ko01100	307	71
Biosynthesis of secondary metabolites	ko01110	194	43
Plant-pathogen interaction	ko04626	93	25
Starch and sucrose metabolism	ko00500	84	8
Amino acid metabolism		67	20
Phenylpropanoid biosynthesis	ko00940	62	16
Microbial metabolism in diverse environments	ko01120	44	14
Cyanoamino acid metabolism	ko00460	30	5
Spliceosome	ko03040	29	20
Plant hormone signal transduction	ko04075	29	5
Flavonoid biosynthesis	ko00941	29	7
Stilbenoid, diarylheptanoid and gingerol biosynthesis	ko00945	29	13
alpha-Linolenic acid metabolism	ko00592	27	5
Diterpenoid biosynthesis	ko00904	23	1
Galactose metabolism	ko00052	21	5
Nitrogen metabolism	ko00910	19	8
Amino sugar and nucleotide sugar metabolism	ko00520	17	1
2-Oxocarboxylic acid metabolism	ko01210	16	4
Fatty acid metabolism	ko01212	15	0
Glucosinolate biosynthesis	ko00966	15	3
Glycerophospholipid metabolism	ko00564	15	0
Viral carcinogenesis	ko05203	13	1
ABC transporters	ko02010	13	1
Cell cycle	ko04110	10	3
Peroxisome	ko04146	10	2
Ras signaling pathway	ko04014	9	0
Biosynthesis of unsaturated fatty acids	ko01040	8	0
Fatty acid biosynthesis	ko00061	8	0
Brassinosteroid biosynthesis	ko00905	7	0
Inositol phosphate metabolism	ko00562	7	1
Glycolysis / Gluconeogenesis	ko00010	6	2
Biosynthesis of amino acids	ko01230	7	6
Fatty acid degradation	ko00071	6	0
RNA transport	ko03013	6	5
Glutathione metabolism	ko00480	6	1
Carbon metabolism	ko01200	5	3
Pyrimidine metabolism	ko00240	5	1
Isoquinoline alkaloid biosynthesis	ko00950	5	1
Drug metabolism—cytochrome P450	ko00982	4	1
DNA replication	ko03030	3	0
Carbon fixation in photosynthetic organisms	ko00710	2	1
Ubiquitin mediated proteolysis	ko04120	1	6
Oxidative phosphorylation	ko00190	0	16

**Fig 3:**
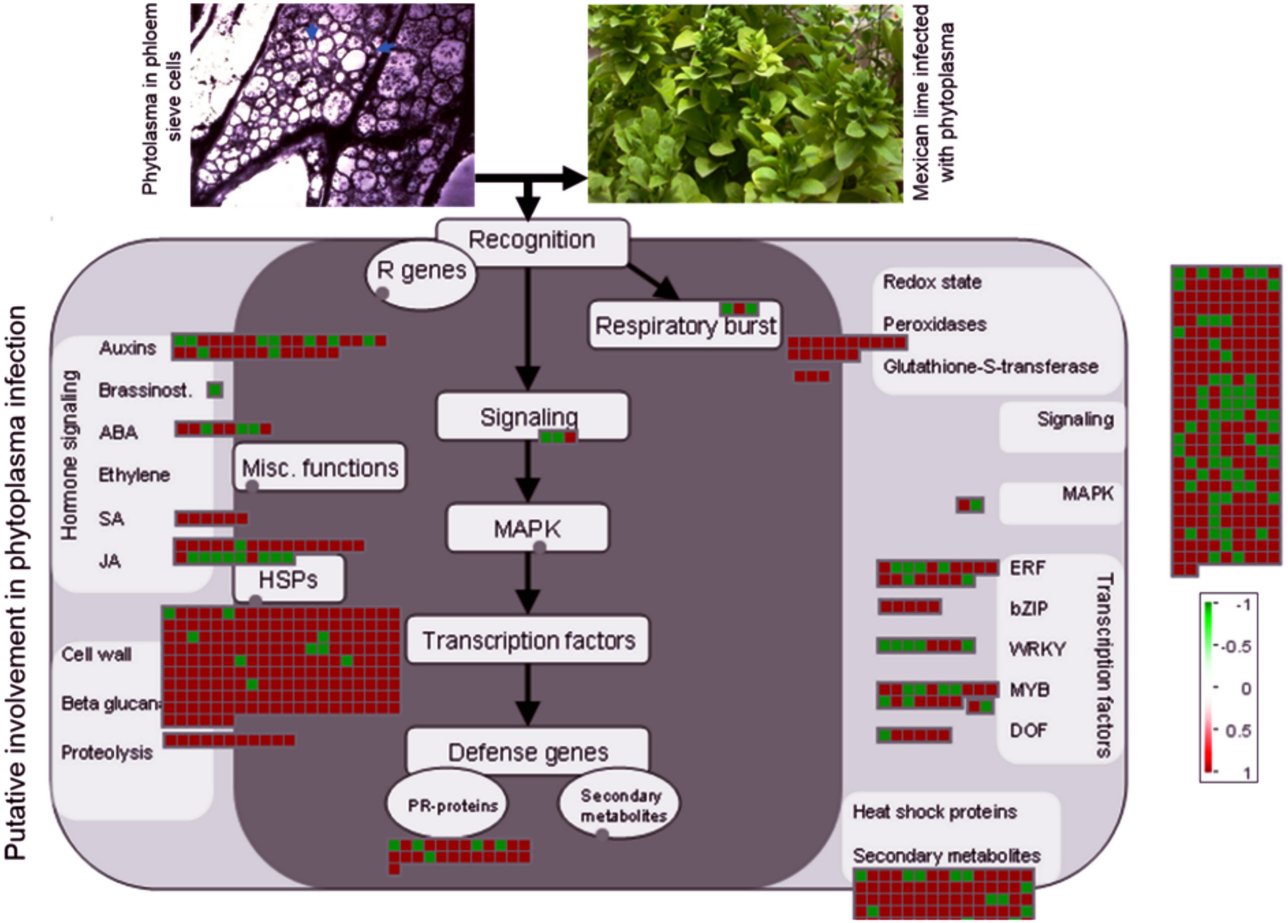
MAPMAN visualisation of differentially expressed unigenes (DEGs) related to metabolism with a log_2_ ratio ≥1.5 between diseased and healthy plants. MAPMAN shows an overview of the cellular response of Mexican lime to phytoplasma infection. A unigene is coloured red if its expression is increased in diseased plants or green if its expression level is decreased in such plants.

Seventy-one DEGs were found to be significantly deregulated (by more than 128-fold); of them, 52 were upregulated and 19 down-regulated in response to phytoplasma infection (Table C in S1 File). Most of these highly deregulated genes were associated with the categories of cell wall, protein synthesis and processing, gene transcription, and mitochondrial electron transport. In addition, MAPMAN software was used to map 2,805 DEGs to metabolic pathways to facilitate interpretation of the results [41]. MAPMAN pathway analysis shows that genes related to cell wall biogenesis and degradation, lipid metabolism, hormone metabolism, transcription, and secondary metabolism are increased in response to phytoplasma infection, while the abundance of transcripts related to oxidative phosphorylation is decreased in diseased plants (Fig 3).

### QRT-PCR confirmation of the expression level of 25 DEGs

**Fig 4:**
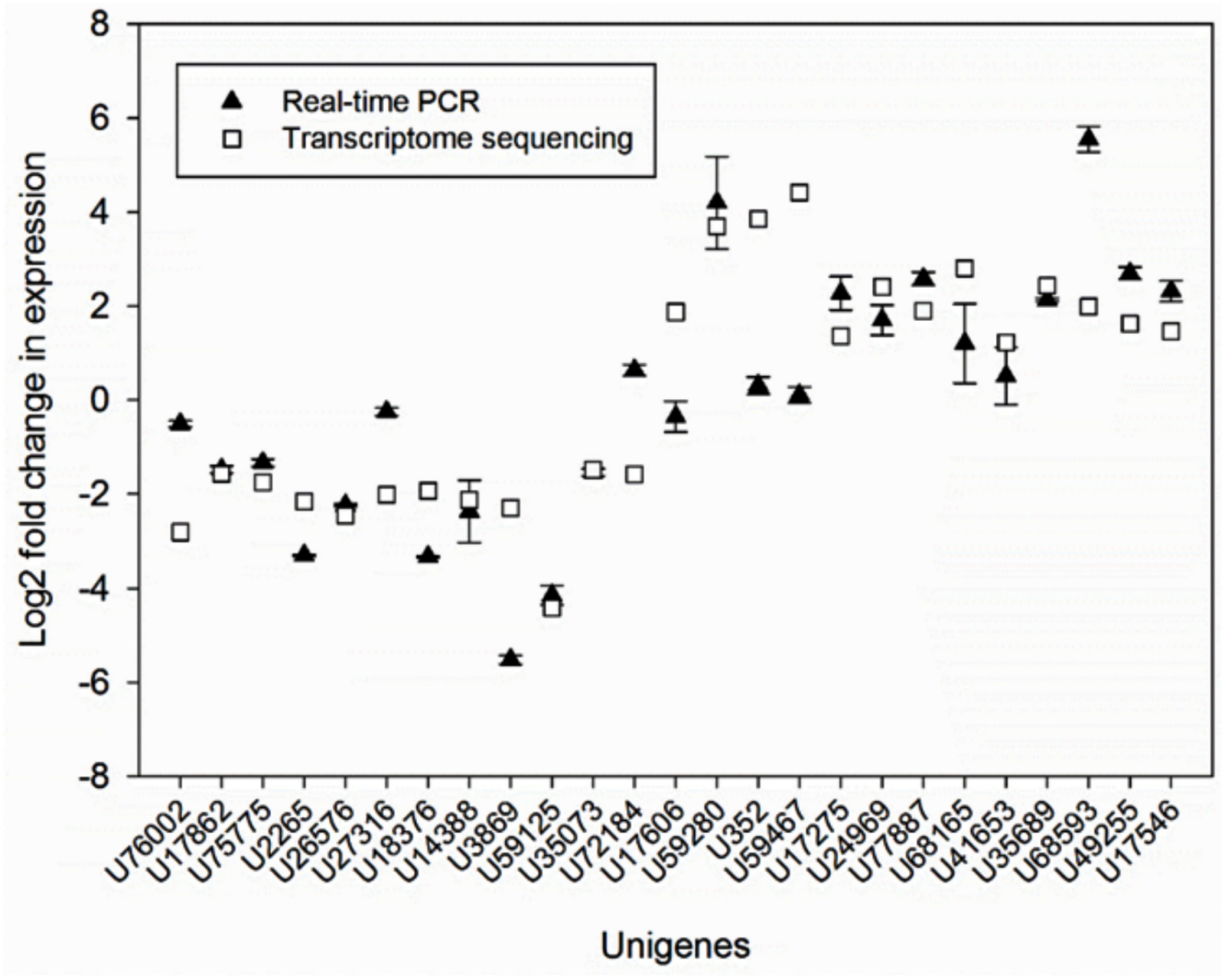
Quantitative real-time RT-PCR (qRT-PCR) confirmation of the differential expression of 25 DEGs in diseased and control plants (U76002; Ammonium transporter, U17862; WRKY transcription factor 21, U75775; Nitrite reductase, U2265; Amino acid transporter, U26576; Mitogen-activated protein kinase kinase kinase 1, U27316; NAC domain-containing protein 71, U18376; Cytochrome P450 84A1, U14388; DELLA protein RGL2, U3869; Jasmonate ZIM domain-containing protein 6, U59125; CRT/DRE binding factor, U35073; Allene oxide synthase, chloroplastic, U72184; Zinc finger A20 and AN1 domain-containing stress-associated protein 3, U17606; BRASSINOSTEROID INSENSITIVE 1-associated receptor kinase 1, U59280; Miraculin-like protein 2, U352; Beta-galactosidase 3, U59467; Proline-rich protein, U17275; phytochrome-interacting factor 3, U24969; Probable LRR receptor-like serine/threonine-protein kinase, U77887; Gibberellin 2-oxidase, U68165; Ent-copalyl diphosphate synthase, U41653; LRR receptor-like serine/threonine-protein kinase GSO1, U35689; Chalcone synthase, U68593; Cyclic nucleotide-gated ion channel 1, U49255; Ent-kaurene oxidase, U17546; Leucine-rich repeat (LRR) protein). The signal intensity of each unigene was normalised against 18S rRNA as a housekeeping gene. The log_2_ fold change in expression of each transcript was determined using the 2^-ΔΔCt^ method and is plotted for comparison with the log_2_ ratios determined using RPKM values in the RNA-seq data. Error bars represent standard error, which is calculated by dividing standard deviation to square root of N. N refers to number of replicate.

In order to evaluate technical and biological variations in our RNA-seq data and to validate our assembly, we further confirmed the differential expression of 25 DEG transcripts (12 down-regulated and 13 up-regulated unigenes) using qRT-PCR analysis. The results of qRT-PCR and the RNA-seq analyses for the selected DEGs are shown in Fig 4. Interestingly, all tested DEGs produced unique PCR products further confirming our assembly approach. In addition, all DEGs showed similar trends in terms of expression, except for U72184 and U17606, the trends of which differed between the two datasets. However, some of the DEGs including U76002, U27316, U18376, U3869, U352, U59467, U17275, U68165, U68593, and U17546 showed significant discrepancies between the qRT-PCR expression values and the RPKM values estimated from RNA-seq data. These discrepancies might have resulted from differences in the mathematical equations used for calculation of the corresponding expression values.

### Cell wall biogenesis and degradation and sucrose metabolism were deregulated in phytoplasma infected plants

Several of the differentially expressed transcripts related to cell wall biogenesis and degradation and sucrose metabolism were up-regulated in diseased plants. These include endoglucanase (8 unigenes), pectin methylesterase (10 unigenes), invertase/pectin methylesterase inhibitor (7 unigenes), cellulase (5 unigenes), glucan 1,3-beta-glucosidase (U9497 and U28080), hydroxyproline-rich glycoprotein (7 unigenes), proline-rich protein (7 unigenes), endo-1,4-beta-xylanase (U21501, U70053, and U64862), beta-1,3-glucanase (6 unigenes), endo-1,4-beta-mannosidase (U1723 and U9643), pectate lyase (13 unigenes), xyloglucan endotransglycosylase (7 unigenes), secondary cell wall-related glycosyltransferase (5 unigenes), polygalacturonase (8 unigenes), expansins (4 unigenes), peroxidase (18 unigenes), pectinesterase (10 unigenes), and chitinase (7 unigenes). Interestingly, significant up-regulation of genes involved in cell wall and carbohydrate metabolism was reported for *Paulownia* infected by witches' broom phytoplasma [27]. Modification of host cell walls might facilitate pathogen infection via the conversion of cell wall polymers into nutritional substrates suitable for pathogen growth and colonisation [42]. Pectin methylesterase (PME) catalyses the removal of methyl esters from cell wall polygalacturonans and consequently changes the charge of the cell wall and enhances the formation of calcium bridges, which might increase cell wall strength [43]. In addition, pectin demethylesterification, which is catalysed by PME, makes pectin susceptible to hydrolysis by polygalacturonase enzymes [44]. Plants express PME-inhibiting proteins to regulate the activity of PME enzymes, which influences the susceptibility of the cell wall to pathogen infection. The overexpression of PME-inhibiting genes (*AtPMEI-1* or *AtPMEI-2*) in *Arabidopsis* was found to enhance pectin methyl esterification and consequently decreased the susceptibility to infection by *Botrytis cinerea* [44]. The levels of eight unigenes that encode polygalacturonases, which affect the texture and strength of demethylesterified cell walls, were significantly increased in diseased plants. In addition, the levels of abundance of four expansin transcripts were also increased in the infected plants. Expansins are auxin-induced proteins that are involved in cell growth via the promotion of cell wall loosening and extension [45].

Endo-1,4-beta-xylanases and endo-1,4-beta-mannosidases catalyse the breakdown of xylan and mannan, respectively, the predominant components of hemicellulose in the cell walls of plants. These enzymes are known to play critical roles in the virulence of plant pathogens, by promoting degradation of the cell walls of host plants [46]. Cellulases and endoglucanases are also directly involved in degradation of the cell walls of plants by breaking down cellulose, the major component of plant cell walls, and are thus considered as pathogenicity-related factors. We also found significant up-regulation of invertase (U30978), sucrose synthase (U18060, U20284, U25544, and U20626), and alpha amylase (U17631) in diseased plants. These enzymes convert sucrose and other carbohydrate oligomers found in phloem sap or released from cell wall polysaccharides into fructose and glucose, which can directly be utilised as sources of energy by phytoplasma. In line with this, significant up-regulation of same genes has been reported in grapevine cultivars infected with Bois noir phytoplasma [47]. Interestingly, transgenic overexpression of a yeast invertase in tobacco and *Arabidopsis* plants increased the content of soluble sugars and starch by the prevention of sucrose export, which resulted in photosynthesis inhibition, stunted growth, and the development of necrosis [48]. Callose (U13633, U23800, and U78075) and cellulose synthase (14 unigenes) showed an increased expression in diseased plants. They are directly involved in cell-wall reinforcement through callose and cellulose deposition, respectively. Interestingly, callose deposition at the phloem sieve plates may further hinder the spread and invasion of the phytoplasma.

It is believed that invading pathogens modify the expression of genes related to cell wall biogenesis and degradation in order to overcome the cell wall barriers and to enhance the release of carbohydrate monomers, which can satisfy their nutritional and energy requirements [42]. In addition, it has been hypothesized that phytoplasmas may alter the expression of cell-wall related enzymes to enhance the release of their effectors into host cells to target host molecules and further facilitate their invasion and multiplication in host cells [49]. Previous reports also showed that, upon phytoplasma infection, the accumulation and translocation of carbohydrates are significantly affected and leaves of phytoplasma-infected plants accumulate higher levels of sugars and starch than disease-free plants [50,51]. The up-regulation of cell wall biogenesis- and degradation-associated proteins in this study is in accordance with the results of our previous proteomic analysis, which showed a higher abundance of a similar set of proteins [14].

### Expression of genes related to plant-pathogen interaction increased in response to phytoplasma infection

Several of the differentially expressed genes are known to be involved in innate immunity and disease responses, such as pathogenesis-related proteins (PR1, PR1a, and PR4b), LRR receptor-like serine/threonine-protein kinase (FLS2, 11 unigenes), disease resistance protein (31 unigenes, RPM1, RPS2, RPS5), serine/threonine-protein kinase PBS1 (13 up-regulated and 1 down-regulated unigenes), thaumatin-like protein (U77950 and U77131), somatic embryogenesis receptor kinase 4 (U76927 and U30814), somatic embryogenesis receptor kinase 1 (14 unigenes), wall-associated receptor (like) kinase (3 down-regulated and 2 up-regulated unigenes), brassinosteroid insensitive 1-associated receptor kinase 1 (BAK1, 5 upregulated unigenes), and cysteine-rich receptor-like protein kinase (7 unigenes). *FLS2* encodes a protein with an extracellular LRR domain (leucine-rich repeat), a transmembrane domain, and an intracellular domain with serine/threonine protein kinase activity [52]. FLS2 binds directly to flagellin as a signal of the presence of pathogenic bacteria and then assembles an active signalling complex, which activates host defence responses [53]. Leucine-rich repeat (LRR) receptor kinase brassinosteroid insensitive 1 (BAK1), a member of the somatic embryogenesis receptor kinases (SERKs), is a central regulator of pathogen-associated molecular pattern (PAMP)-triggered immunity [53]. Most pattern recognition receptors interact with BAK1 to activate defence responses that form part of innate immunity. Similarly to FLS2, LRR receptor-like serine/threonine-protein kinase (EFR) and chitin elicitor receptor kinase 1 (CERK1) are pattern recognition receptors, which bind to EF-Tu and chitin, respectively, as signals of the presence of bacterial and fungal pathogens and activate downstream innate immunity responses [53,54]. Wall-associated kinases (WAKs) are membrane-bound receptors that associate with pectin in the cell wall and are known to be involved in cell elongation and immune response [55]. They are induced in response to wounding and pathogen infection, and a recent report has shown that the overexpression of WAK1 in *Arabidopsis* increased resistance to *B*. *cinerea* [56].

The level of cyclic nucleotide gated channel (CNGC, 7 unigenes) significantly increased in response to phytoplasma infection. CNGCs are non-selective cation channels that are thought to play an essential role in defence against pathogens, development, and ion homeostasis [57]. Upon pathogen recognition, CNGCs mediate increased influx of cations such H^+^ and Ca^2+^, which in the case of Ca^2+^ is associated with defence responses, oxidative burst, and finally pathogen-induced cell death (hypersensitivity reaction), which is an extremely effective approach to combat with biotrophic pathogens such as phytoplasma [57,58]. Deregulation of CNGC transcripts has also been reported in witches’ broom disease in *Paulownia* [28]. The increased cytosolic Ca^2+^ is sensed by Ca^2+^-binding proteins (calmodulins, 2 unigenes with increased abundance in infected plants), which transduce the elevated Ca^2+^ signal to downstream signalling pathways, leading to an appropriate defence response to pathogen infection [59].

The innate immunity response that is activated upon pathogen recognition is usually accompanied by transcriptional reprogramming and the induction of defence-related genes. Several of the differentially expressed genes were found to be final transcriptional regulators of innate immunity responses, such as JAZ, the WRKY family of transcription factors (10 unigenes that were up-/down-regulated), and MYC2 (6 unigenes). Transcription factors of the WRKY family are known as positive and negative regulators of innate immunity responses [60]. JAZ repressors, the levels of which were significantly decreased in phytoplasma-infected plants, act as negative regulators of MYC2 [61]. MYC2 is a transcriptional activator that binds to the conserved G-box present in the promoters of JA-responsive genes and therefore activates defence-related genes [61].

### Genes related to several pathways for the biosynthesis of secondary metabolites were deregulated in response to phytoplasma infection

The phenylpropanoid biosynthesis pathway contributes to the production of lignin, suberin, and condensed tannins as well as other secondary metabolites that are essential for the responses to various biotic and abiotic stimuli [62,63]. Several transcripts related to this pathway, including 4-coumarate-CoA ligase 1 (4CL, 2 unigenes), cinnamyl-alcohol dehydrogenase (2 unigenes), phenylalanine ammonia-lyase (PAL, 5 unigenes), coniferyl-alcohol glucosyltransferase (1 unigene), isochorismate synthase 1 (1 unigene), and shikimate O-hydroxycinnamoyltransferase (15 up-regulated and 7 down-regulated unigenes), were deregulated in response to phytoplasma infection in diseased plants. PAL catalyses the deamination reaction of phenylalanine to cinnamic acid, which is the flux control point of the phenylpropanoid pathway [63]. 4CL, the last enzyme of the general phenylpropanoid pathway, catalyses the formation of CoA thiol esters of 4-coumarate and other hydroxycinnamates, which serve as intermediates for lignin biosynthesis. In addition, we found significant up-regulation of genes that encode enzymes that mediate the final steps of the biosynthesis of lignin precursors (Figure E in S2 File). A similar set of genes have been reported to be up/down-regulated in cotton plants in response to the fungal pathogen *Verticillium dahlia*, which suggests that lignin metabolism plays a critical role in resistance to pathogen infection [25].

Several DEGs including naringenin 3-dioxygenase (2 unigenes), chalcone synthase (CHS, 4 unigenes), leucoanthocyanidin dioxygenase (3 unigenes), and flavonoid 3'-monooxygenase (7 unigenes) were found to be involved in the flavonoid biosynthesis pathway, which utilises phenylpropanoid derivatives to produce flavonoids. The combined action of these two biosynthetic pathways results in the production of secondary metabolites, such as stilbenes, coumarins, and isoflavonoids, which are phytoalexins produced by diseased plants, and acetosyringone and salicylic acid (SA), which are involved in plant-pathogen interactions [64]. Chalcone synthase (CHS) is a key enzyme in the flavonoid/isoflavonoid biosynthetic pathway, which provides the starting materials for a diverse set of secondary metabolites with important roles in plant defence [65]. PAL and isochorismate synthase are drivers of SA biosynthesis, which is well known to be involved in plant-pathogen interaction.

**Fig 5:**
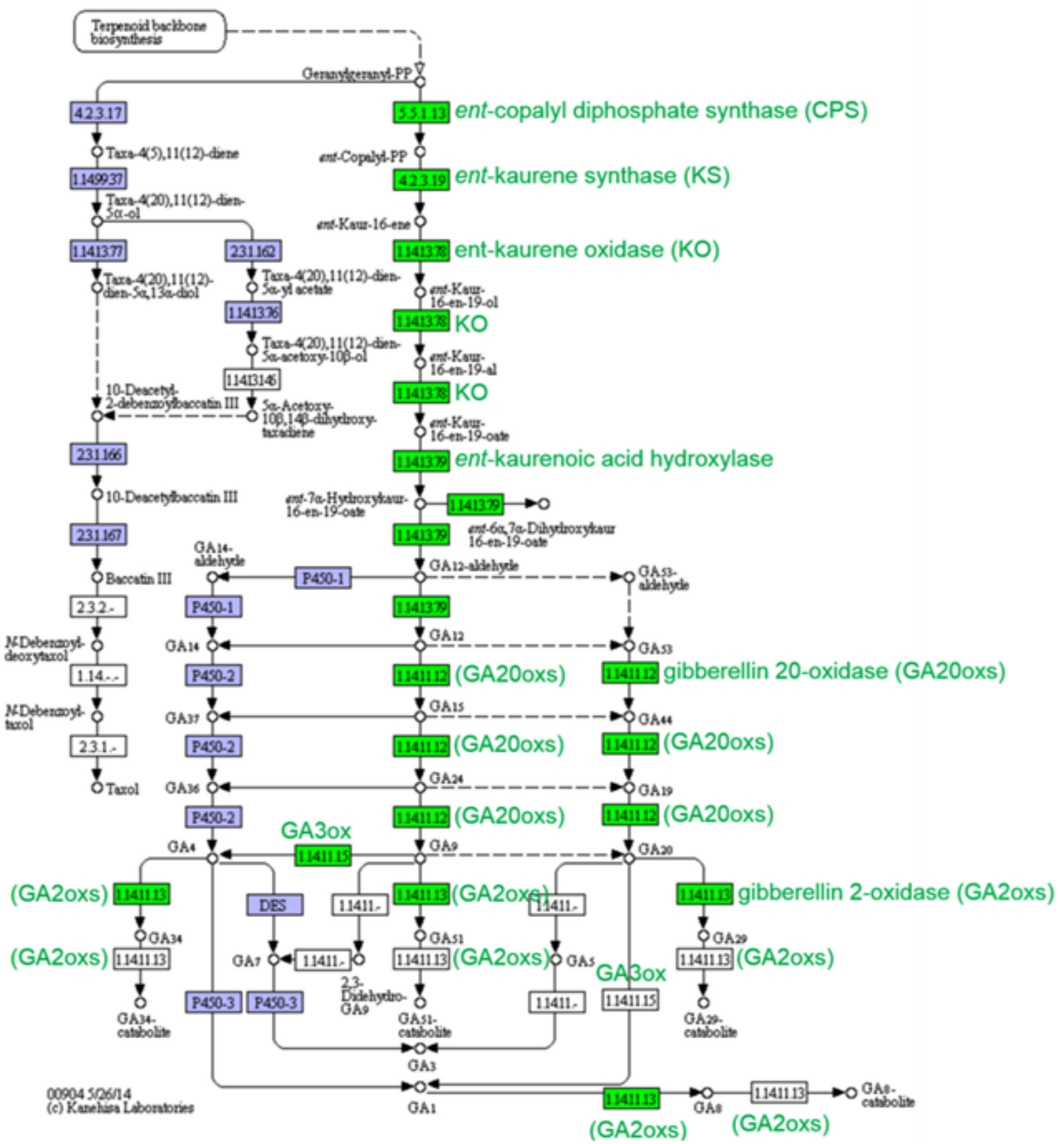
Gibberellin (GA) biosynthesis pathway. GAs are synthesised using granylgranyl diphosphate as a precursor in the phenylpropanoid biosynthesis pathway. In our study, genes related to the GA biosynthesis pathway were coordinately up-regulated in phytoplasma-infected plants. In addition, the GA-catabolising enzyme gibberellin 2 oxidase (GA20oxs), which inactivates bioactive GAs and therefore reduces their cellular level, was also significantly overexpressed in diseased plants. Enzymes coloured green are those that were up-regulated, whereas enzymes shown in blue or white without a green label are those that were not identified in the present study.

Terpenoid metabolites are known as signalling molecules involved in many interactions of plants with other organisms, including pathogens. For example, certain diterpenoid and sesquiterpene metabolites are produced as plant growth regulators (gibberellins, GAs) and phytoalexins in response to pathogen infection [66]. In our study, genes related to the diterpenoid pathway, which leads to the biosynthesis and catabolism of GAs, were up-regulated by phytoplasma infection (Fig 5). These include ent-copalyl diphosphate synthase (CPS, 4 unigenes), ent-kaurene synthase (KS, 2 unigenes), ent-kaurene oxidase (KO, 2 unigenes), ent-kaurenoic acid oxidase 2 (KAO, 5 unigenes), ent-kaurenoic acid hydroxylase (6 unigenes), gibberellin 3-beta-dioxygenase (2 unigenes), gibberellin 2-oxidase (GA2oxs, 2 unigenes), gibberellin 20 oxidase (GA20oxs, 1 unigene), 2-oxoglutarate-dependent dioxygenase (4 unigenes), and casbene synthase (7 unigenes). The biosynthesis of GAs is initiated by the conversion of geranylgeranyl diphosphate, a common precursor of diterpenoids, to copalyl diphosphate in a reaction catalysed by CPS, which is then further cyclised to ent-kaurene by the enzymatic action of KS (Fig 5). Ent-kaurene is oxidised to ent-kaurenoic acid in a reaction catalysed by KO, a membrane-associated cytochrome P450 monooxygenase, and then converted to GA12 by KAO. GA12 is further converted to GA53 by 13-hydroxylation. Finally, GA12 and GA53 are converted to various bioactive GAs by a series of oxidation steps catalysed by gibberellin oxidases (GA20oxs, 2-oxoglutarate-dependent dioxygenase, and GA3oxs) [67]. In contrast, GA2oxs inactivates bioactive GAs and is therefore responsible for a reduction in the level of bioactive GAs in plants. It has been shown that rice mutants overexpressing GA2oxs show early and increased tiller, a semidwarfism phenotype, and adventitious root growth [68]. In addition, change in the level of bioactive GAs may also affect resistance to pathogen infection. For example, recently, it has been shown that overexpression of a GA-deactivating enzyme, elongated uppermost internode (EUI), in rice decreased the GA level and increased resistance to bacterial and fungal pathogens, which suggests a negative role for GAs in the development of resistance against pathogen infection [69].

CPS and KS are key enzymes that catalyse the first committed step of the GA biosynthesis pathway; their expression is tightly regulated at the transcriptional level [67,70]. CPS has also been shown to be induced in response to the pathogen *Fusarium* in maize [71]. In addition to being involved in the biosynthesis of GAs, some CPSs are known to participate in the biosynthesis of phytoalexin diterpenoids. GAs are diverse diterpenoid compounds that are involved in several developmental processes including seed germination, stem elongation, leaf expansion, and flower and fruit development [67,72]. The precise role of GAs in the development of defence responses against pathogens is unknown; however, evidence suggests that GA signalling pathways may be involved in plant-pathogen interaction [73]. Accordingly, we found significant down-regulation of gibberellin receptor GID1 (4 unigenes) and DELLA protein RGL2 (1 unigene) and up-regulation of phytochrome-interacting factor 3 (PIF3, 2 unigenes) in response to phytoplasma infection, which further confirmed using qRT-PCR. In the absence of GA, DELLA protein, a negative regulator of GA signalling, binds and inactivates phytochrome interacting factors (PIFs), a class of helix-loop-helix transcription factors [72]. When GA is present, it binds GID1, causes its conformational change, and facilitates its binding to DELLA. The formation of GID1-DELLA complex triggers DELLA ubiquitination and degradation by 26S proteasome [72]. Degradation of DELLA enhances the release of PIFs and promotes the induction of GA-responsive genes. It has been shown that DELLA proteins may also modulate plant immunity responses by controlling SA- and JA-dependent defence responses. Interestingly, *Arabidopsis* plants deficient in DELLA proteins show enhanced resistance to biotrophic pathogens Pst DC3000 and *Hyaloperonospora arabidopsidis* [74]. In addition, emerging evidence suggests that mutation in GA-perceiving protein (GID1) may also affect defence responses. For example, rice mutants defective in GID1 accumulate GA at an increased level and show enhanced resistance to the fungal pathogen *Magnaporthe grisea*, which suggests a negative role for GID1 in the establishment of resistance against pathogen infection [73]. Increased expression of GA biosynthesis-related genes, such as GA 3-β hydroxylase, GA20oxs, and GA2oxs, was also reported in cotton plants infected by *Verticillium dahlia* [25]. In our opinion, the deregulation of genes related to the GA biosynthesis and signalling pathways suggests that GA might be a key hormone that contributes to the development of witches’ broom symptoms, which is typically accompanied with suppressed stem elongation and leaf expansion.

### Genes related to oxidative phosphorylation were down-regulated in diseased plants

We identified significant down-regulation of key genes related to oxidative phosphorylation in the infected plants. These include ubiquinol-cytochrome c reductase (2 unigenes), F-type H+-transporting ATPase (1 unigene), cytochrome c oxidase (6 unigenes, subunits I, II, III), NADH-ubiquinone oxidoreductase (6 unigenes), and NADH dehydrogenase (6 unigenes). This may imply the cessation of processes for the production of plant energy in response to phytoplasma infection.

### Change in the biosynthesis and signalling pathways related to the plant hormones JA, auxin, ethylene, and BRs in phytoplasma-infected plants

Analysis of DEGs during phytoplasma infection in Mexican lime trees revealed that several genes related to pathways for hormone biosynthesis and signalling were deregulated. Plant hormones are known to play a critical role in the development of defence responses against pathogen infection. Lipoxygenase (LOX1 and 2, 20 unigenes) and allene oxide synthase (AOS, 3 down-regulated unigenes) are enzymes involved in the biosynthesis of jasmonic acid and its derivatives. In an array of enzymatic reactions, linolenic acid is oxygenated by LOX and the resulting 13-hydroperoxide is converted to an allene oxide by the enzymatic action of AOS, which is the first committed step of JA biosynthesis [75]. Allene oxide is finally converted to cyclopentanone acids, which are direct precursors of jasmonates. Jasmonate O-methyltransferase (5 unigenes) that was increased in response to phytoplasma infection, catalyses the methylation of jasmonate to methyl jasmonate. Down-regulation of AOS, a key enzyme in the JA biosynthesis pathway, may further hinder the production of JA. It is believed that JA does not mediate defence responses against biotrophic pathogens (such as phytoplasma); instead, it plays an important role in defence against necrotrophic pathogens [72] and insects that may serve as phytoplasma vectors [76]. In addition, we found significant down-regulation of jasmonate-ZIM-domain containing proteins 1, 6, and 8, which suppress the JA signalling pathway by inhibiting the expression of JA-responsive genes. Down regulation of genes related to the JA biosynthesis and signalling pathways in phytoplasma infected plants increases the fitness of phytoplasmas insect vectors and therefore facilitate the spread of phytoplasmas [49].

Some DEGs were found to being involved in the biosynthesis and signalling of the plant hormone auxin. These include indole-3-acetic acid-amido synthetase (IAA amido synthetase, 2 unigenes), SAUR family protein (5 unigenes), auxin efflux carrier family protein (4 unigenes), AUX/IAA protein (1 up-regulated unigene), auxin-responsive protein (4 unigenes), and auxin-induced protein (11 up-regulated and 2 down-regulated unigenes). Studies have demonstrated that auxin is involved in disease susceptibility, and the suppression of auxin signalling using microRNAs that target auxin receptors was shown to induce an immune response in *Arabidopsis* [77]. An elevated auxin level is usually accompanied by increased susceptibility to biotrophic pathogens [72]. Auxin induces the expression of expansins (4 up-regulated unigenes), which are involved in cell wall loosening and growth, and consequently promote disease susceptibility. We found significant up-regulation of IAA amido synthetase, which prevents free IAA accumulation and therefore enhances disease resistance [78]. AUX/IAA family proteins suppress auxin-responsive genes through binding and inactivating auxin response factors, which are positive regulators of the auxin signalling pathway [72]. In addition, increased expression of auxin-inducible SAUR family proteins was also noted in diseased plants. SAUR proteins comprise a large family of auxin-responsive proteins, the exact function of which is unknown. However, recent evidence suggests that they may be involved in cell expansion through modulating auxin transport [79].

Significant up/down-regulation of genes related to the ethylene signalling was also detected in diseased plants including AP2-like ethylene-responsive transcription factor (1 up-regulated unigene), ethylene-responsive transcription factor (6 down-regulated and 10 up-regulated unigenes), and ethylene-induced esterase (3 up-regulated unigenes). The role of ethylene in plant-pathogen interaction is complex and depends of the plant species and the type of pathogen; it may either enhance disease susceptibility or increase disease resistance [80].

Brassinosteroids (BRs) are plant growth regulators involved in developmental processes and responses to abiotic and biotic stresses. The exogenous application of BRs increases resistance to a wide range of biotrophic pathogens [72,81], which suggests their important role in plant responses to pathogen infection. Interestingly, we found significant up-regulation of genes related to the biosynthesis and signalling of BRs. These include steroid 22-alpha-hydroxylase (3 unigenes), brassinosteroid-6-oxidase 1 (2 unigenes), 3-epi-6-deoxocathasterone 23-monooxygenase (2 unigenes), and cytochrome P450, family 724, subfamily B (2 unigenes). In addition, 19 up-regulated unigenes were identified as protein brassinosteroid insensitive 1 (BRI1) and 5 unigenes as brassinosteroid insensitive 1-associated receptor kinase 1 (BAK1). BRs are synthesised by a complex array of hydroxylation reactions catalysed by cytochrome P450 family enzymes. BRI1 binds and perceives brassinolids (BLs, bioactive BRs) at the cell surface. BL binding activates the kinase activity of BRI1 and promotes the phosphorylation and dissociation of BAK1, which further activates downstream BR-dependent signalling pathways [72]. As noted above, BAK1 also plays a critical role in the establishment of innate immunity responses. However, it should be noted that the role of BAK1 in such responses is BR-independent, further suggesting the dual role of BAK1 in the regulation of developmental processes and defence responses [81]. BRs are known to modulate the expression of genes related to defence against pathogen infection, presumably through the induction of PR1 expression and biosynthesis of the plant hormone SA [72,81].

### Conclusion

In summary, using NGS we sequenced the transcriptome of Mexican lime trees infected with Ca. *P*. *aurantifolia*, the causative agent of witches’ broom disease, and that of healthy plants to explore the change in gene expression with the aim of discovering molecular mechanisms behind phytoplasma pathogenicity. Using 2×54,177,778 paired end reads we could successfully assemble and recover 78,185 unigenes. More than 53% of unigenes could successfully be annotated against the NCBI nr database. The remaining unannotated sequences were either species specific or might be truncated transcripts originated from non-conserved or untranslated regions of genes. Comparing the transcript abundances of annotated unigenes between healthy and infected plants showed the differential abundance (over 2.8-fold) of 2,805 unigenes. These differentially expressed unigenes were significantly enriched in 43 KEGG metabolic and regulatory pathways. Interestingly, our results showed a significant and coordinated up-regulation of genes related to the pathway for the biosynthesis of plant hormone GA in phytoplasma infected plants. In addition, an increased abundance of genes related to cell-wall biogenesis and degradation, secondary metabolism, and innate immunity were observed in diseased plants. However, genes related to the pathway for ATP biosynthesis processes and ubiquitin-mediated proteolysis were co-ordinately down-regulated in diseased plants. The findings of this study can be valuable for unravelling the molecular mechanisms of plant-phytoplasma interactions and can pave the way for engineering lime trees with resistance to witches’ broom disease.

## Supporting Information

S1 File **Table A in S1 File.** The sequence of primers used for real-time PCR confirmation of the expression of some of the candidate up/down regulated DEGs. **Table B in S1 File:** Number and length distribution of contigs, scaffolds and unigenes assembled in healthy and infected libraries. **Table C in S1 File:** Differentially expressed unigenes with a significantly up/down regulation (more than 128-fold).
(DOCX)

S2 File **Figure A in S2 File:** (A) Distribution of the length of unigenes in infected (I), healthy (H), and pooled libraries (All). A significant proportion of unigenes were between 200 and 300 nt in length, with most of them remaining unannotated. (B) The number of gaps in unigenes. The gap distribution represents the number of N divided by the sequence length of the assembled unigene. **Figure B in S2 File:** Species (A and B) and e-value (C and D) distribution of top blast hits from the NCBI non-redundant protein (nr) and Swiss-Prot databases, respectively The majority of unigene were matched with significantly low e-values to sequences from species with well-annotated genomes. **Figure C in S2 File:** GO functional classification. A total of 18,336 unigenes (23% of total unigenes) were assigned to at least one GO term and classified into the three high-level GO terms: biological processes, cellular components, and molecular functions. **Figure D in S2 File:** Classification of unigenes based on Cluster of Orthologous Group (COG) of genes. A total of 11,736 unigenes that produced significant hits when probed against the NCBI nr database were classified into 25 functional categories. **Figure E in S2 File:** KEGG pathway visualisation of differentially expressed unigenes related to the phenylpropanoid biosynthesis pathway. Up-regulated enzymes are coloured green and down-regulated enzymes are coloured red. The majority of enzymes (peroxidase, coniferyl-alcohol glucosyltransferase, and caffeic acid 3-O-methyltransferase) that catalyse the final steps of the biosynthesis of lignin precursors (syringin, syringyl lignin, guaiacyl lignin, 5-hydroxy guaiacyl lignin) were up-regulated in diseased plants.
(DOCX)
