# Supplementary material for: In-Depth Transcriptome Sequencing of Mexican Lime Trees Infected with Candidatus Phytoplasma aurantifolia
Source: PLoS One. 2015 Jul 1;10(7):e0130425. doi: 10.1371/journal.pone.0130425 (PMC4489016; doi:10.1371/journal.pone.0130425)
Supplement: S1 File — Table A in S1 File. The sequence of primers used for real-time PCR confirmation of the expression of some of the candidate up/down regulated DEGs. Table B in S1 File: Number and length distribution of contigs, scaffolds and unigenes assembled in healthy and infected libraries. Table C in S1 File: Differentially expressed unigenes with a significantly up/down regulation (more than 128-fold). (DOCX) [file pone.0130425.s001.docx]

Table A

|  | UnigenesID | Forward primer | Reverse primer | Annealing temperature | Amplicon length |
| --- | --- | --- | --- | --- | --- |
| 1 | U59467 | CGGTCCCTTTTGTTTTGAAC | AGGCGGTTGAGGTAGTTG | 56 | 138 |
| 2 | U352 | TGGCTCTGGATGGCATTG | GTGCTTCTGGGATAGTGA | 56 | 133 |
| 3 | U59280 | CATTCATTACGCGGTCATTG | CCGGCTTTGAATGTTCTG | 55 | 120 |
| 4 | U18376 | CTCACTAGGGCTCACTCCAT | ATGCCCCAGACTTCACAG | 60 | 164 |
| 5 | U2265 | TGCTGCATTGGTTCTGTC | GACTGCAAAGGACTCCAAG | 58 | 130 |
| 6 | U17862 | GACTCCGGCTTATTCCATG | GATAGGTCATCGGGAAGCA | 57 | 130 |
| 7 | U27316 | ATGCGATACACAACCCAATCT | CGGCCATGAGACCAAAACT | 60 | 126 |
| 8 | U75775 | GAAGGAGCTGACGTTTTC | CTTCTGCCTCTTCCCTCTC | 60 | 160 |
| 9 | U76002 | TTGCCGGCTCTATTCTTCTC | CCGCTCTTTTATGGGCTC | 59 | 163 |
| 10 | U26576 | GATTGTCCGCCCAGTAGTG | CACGCGATCAGCCAAACTC | 60 | 174 |
| 11 | U72184 | CAAAGAGATGGGCAAAGAG | GCCAAATTACAAACCAAACGA | 56 | 121 |
| 12 | U35073 | TCAGTGGAGGGCATGTAG | TTTCGTGCCAACATGCCC | 52 | 149 |
| 13 | U17546 | GAGAACAAGCATTGAGCAC | TCACTTTCCATCAAGACCC | 58 | 200 |
| 14 | U59125 | TATGGGGATAAGGGGTGT | TGCCACAACTAACCTCCTC | 60 | 182 |
| 15 | U35689 | TCTCATTTTCTGCTCACTT | GGTTGTTTTCAGCAAAGT | 50 | 168 |
| 16 | U68165 | CTGCTGAGATTACATGGTT | CTCTTCAGGGAATTGCAC | 62 | 147 |
| 17 | U68593 | GACTCTCTTTCAATGCCA | TTGAAAGCACAGGTTCCGA | 60 | 119 |
| 18 | U49255 | TAAATTGGTCCGTACACC | AATTAGCCGTTAATCTTCC | 50 | 132 |
| 19 | U77887 | CATGCCATCCTCTTCACT | GGGTTGGGTTGAGTATCT | 58 | 123 |
| 20 | U3869 | CTCCTCCTCCTCCTCCAAAG | GCGAACCCATCACACTACAT | 60 | 117 |
| 21 | U17275 | AACACCCATTTGCATTCTC | GGTTTGTATGCCTTCGATG | 58 | 130 |
| 22 | U41653 | GAGAGTAGCAAGACCTCAAG | TATCACCAGCCTCACTTCAC | 62 | 114 |
| 23 | U14388 | AGCAAACACGGATGACAAGG | TGCCTCAGCCACATTTTCCA | 60 | 149 |
| 24 | U17606 | CTCACCGCAGATTTTGAACCAC | ACATCCGTCTTCTCATCCACA | 60 | 158 |
| 25 | U24969 | GCCTCCGTTTCCAATTCTC | GATACCGAGGATTTCATGGC | 62 | 131 |

Table B

|  | Healthy | | | Infected | | |  |
| --- | --- | --- | --- | --- | --- | --- | --- |
|  | contigs | scaffolds | unigenes | contigs | scaffolds | unigenes | All unigenes |
| 100-500 nt | 448,140 | 107,978 | 63,739 | 569,063 | 121,506 | 71,324 | 54,336 |
| 500-1000 nt | 10854 | 11,849 | 11,898 | 10990 | 12,390 | 12,431 | 15,479 |
| 1000-1500 nt | 2191 | 2,897 | 2,918 | 1951 | 2,719 | 2,739 | 4,512 |
| 1500-2000 nt | 674 | 1,133 | 1,155 | 570 | 1,026 | 1,031 | 2,049 |
| >=2000 nt | 303 | 891 | 897 | 257 | 772 | 784 | 1,809 |
| total | 462,162 | 124,748 | 80,607 | 582,831 | 138,413 | 88,309 | 78,185 |
| N50 | 126 | 397 | 485 | 103 | 369 | 449 | 648 |
| average length (nt) | 142 | 305 | 407 | 133 | 291 | 388 | 530 |
| total nucleotides length (nt) | 65,616,496 | 38,061,279 | 32,805,334 | 77,337,326 | 40,271,775 | 34,281,386 | 32,960,632 |

Table C

| **Gene** | **Annotation** | ***p*-value** | **Log2 Fold change** |
| --- | --- | --- | --- |
| **Cell wall biogenesis and degradation** | | | |
| U73568 | COBL8 (COBRA-LIKE PROTEIN 8 PRECURSOR) [Arabidopsis thaliana] | 0.02312 | 7.126704 |
| U60555 | lyase [Arabidopsis thaliana] | 0.00096 | 7.402756 |
| U76363 | COBRA-like protein [Eucalyptus nitens] | 3.09E-06 | 8.466097 |
| U71221 | transferase, transferring glycosyl groups [Arabidopsis thaliana] | 0.00014 | 8.560906 |
| U76850 | pectatelyase [Carica papaya] | 2.22E-16 | 8.639305 |
| U61873 | AT4g24430/T22A6_260 [Arabidopsis thaliana] | 2.53E-12 | 8.838763 |
| U76436 | glycosyltransferase [Populustrichocarpa] | 2.22E-16 | 11.22039 |
| **Plant-pathogen interaction** | | | |
| U63098 | disease resistance-responsive family protein [Arachishypogaea] | 0.00096 | 7.995371 |
| U57910 | pathogenesis-related protein 1a [Malus x domestica] | 0.00108 | -7.60925 |
| U76770 | basic chitinase [Nicotianatabacum] | 0.00051 | 7.606886 |
| U75273 | chitinase 134 [Nicotianatabacum] | 8.64E-07 | 7.709256 |
| U63098 | disease resistance-responsive family protein [Arachishypogaea] | 0.00096 | 7.995371 |
| **Gene transcription** | | | |
| U75424 | helix-loop-helix DNA-binding protein-like [Oryza sativa Japonica Group] | 0.00647 | 7.321838 |
| U75144 | OBP3-responsive gene 3 [Arabidopsis lyrata subsp. lyrata] | 0.00181 | 7.684117 |
| U59782 | AtGRF5 (Growth-Regulating Factor 5); transcription activator [Arabidopsis thaliana] | 0.02312 | 7.210233 |
| U72665 | SNF2 domain-containing protein [Arabidopsis lyrata subsp. lyrata] | 0.02312 | 7.216358 |
| U75424 | helix-loop-helix DNA-binding protein-like [Oryza sativa Japonica Group] | 0.006 | 7.321838 |
| U75144 | OBP3-responsive gene 3 [Arabidopsis lyrata subsp. lyrata] | 0.00181 | 7.684117 |
| U76627 | binding [Arabidopsis thaliana] | 0.00027 | 7.767522 |
| U75261 | MYB20 [Malus x domestica] | 7.48E-05 | 8.2538 |
| U76525 | tubby-like protein 13 [Oryza sativa Japonica Group] | 1.63E-06 | 8.488925 |
| U47388 | pentatricopeptide repeat protein [Porellaplatyphylla] | 5.57E-06 | -7.68464 |
| U54917 | AtMYB103/AtMYB80 [Arabidopsis lyrata subsp. lyrata] | 0.00229 | -7.86078 |
| U74105 | zinc finger family protein [Arabidopsis lyrata | 0.01223 | 7.297833 |
| **Signaling** | | | |
| U75799 | calmodulin [Phytophthorainfestans T30-4] | 7.48E-05 | 8.16546 |
| U59824 | catalase [Nicotianasylvestris] | 9.99E-09 | 8.029812 |
| **Protein synthesis and processing** | | | |
| U71426 | ribosomal protein L27 [Phytophthorainfestans T30-4] | 0.04373 | 7.0621 |
| U71088 | RL14, ribosomal protein 14 60S large ribosomal subunit [Thalassiosirapseudonana CCMP1335] | 0.04373 | 7.082149 |
| U69866 | 60S ribosomal protein L5 [Phytophthorainfestans T30-4] | 0.04373 | 7.16541 |
| U73135 | ribosomal protein L34 [Phytophthorainfestans] | 0.00647 | 7.582857 |
| U76758 | ribosomal protein L2 [Pseudendocloniumakinetum] | 7.48E-05 | 7.936932 |
| U74587 | elongation translation factor 1 alpha [Cyanophoraparadoxa] | 0.02312 | 7.020591 |
| U73604 | EEF1A2, eukaryotic translation elongation factor 1 alpha [Ectocarpussiliculosus] | 0.01223 | 7.343319 |
| U75339 | elongation factor EF-2 [Paulinellachromatophora] | 0.00342 | 7.501758 |
| U67709 | ubiquitin-associated /TS-N domain-containing protein [Arabidopsis lyrata subsp. lyrata] | 0.00181 | 7.94714 |
| U74605 | methionine aminopeptidase 1B [Zea mays] | 3.95E-05 | 8.430285 |
| U60536 | virulence-related protein Nf314 [Zea mays] | 3.57E-08 | 8.702034 |
| U54808 | 60S ribosomal protein L30 [Arabidopsis lyrata subsp. lyrata] | 0.02189 | -7.18913 |
| U54172 | ribosomal protein L37ae [Micromonas sp. RCC299] | 0.02189 | -7.24859 |
| U44845 | RL22, ribosomal protein 22 60S large ribosomal subunit [ThalassiosirapseudonanaCCMP1335] | 0.01031 | -7.58067 |
| U56998 | Ribosomal protein S27 [Ectocarpussiliculosus] | 0.00108 | -7.77735 |
| U56690 | 40S ribosomal protein S11 [Cyanophoraparadoxa] | 0.00108 | -7.8157 |
| U44367 | component of cytosolic 80S ribosome and 60S large subunit [Volvoxcarteri f. nagariensis] | 0.00229 | -7.96769 |
| U57526 | 60S ribosomal protein L38 | 0.00024 | -7.98288 |
| U58103 | elongation translation factor 1 alpha [Cyanophoraparadoxa] | 0.00108 | -7.56659 |
| **photosynthesis** | | | |
| U60364 | ferredoxin--NADP(+) reductase [Nicotianatabacum] | 0.01223 | 7.144148 |
| U75392 | chloroplast Tsip1-interacting ferredoxin [Nicotianatabacum] | 0.00181 | 7.648753 |
| U64285 | major chlorophyll a/b binding protein LHCb1.1 [Spinaciaoleracea] | 0.00647 | 7.662562 |
| U75389 | light-harvesting chlorophyll a /b binding protein [Nicotianatabacum] | 0.00096 | 7.78627 |
| U74795 | NtpII10 [Nicotianatabacum] | 3.09E-06 | 8.730912 |
| U49187 | apocytochrome b [Emilianiahuxleyi] | 2.74E-07 | -8.32472 |
| **Calvin cycle** | | | |
| U59991 | ribulose-1,5-bisphosphate carboxylase small subunit [Nicotianatabacum] | 2.22E-16 | 10.28795 |
| U71840 | plastidicaldolase [Solanumtuberosum] | 0.02312 | 7.285772 |
| U76868 | glyceraldehyde-3-phosphate dehydrogenase A-subunit precursor [Nicotianatabacum] | 0.00181 | 7.307975 |
| U73593 | plastidicaldolase NPALDP1 [Nicotianapaniculata] | 0.00647 | 7.535975 |
| U62043 | ribulosebisphosphate carboxylase activase [Nicotianatabacum] | 2.22E-16 | 8.435504 |
| **photorespiration** | | | |
| U73511 | glycolate oxidase [Nicotianatabacum] | 0.02312 | 7.132474 |
| **Nucleotide metabolism** | | | |
| U70100 | inosine-uridine preferring nucleoside hydrolase family protein [Arabidopsis thaliana] | 0.04373 | 7.15127 |
| U77160 | uridylate kinase [Paulinellachromatophora] | 4.13E-10 | 8.941752 |
| **Secondary metabolism** | | | |
| U75976 | orcinol O-methyltransferase 4 [Rosa hybrid cultivar] | 0.00027 | 7.926059 |
| **Polyamine metabolism** | | | |
| U66150 | putrescine N-methyltransferase 1 [Nicotianaattenuata] | 1.63E-06 | 7.418359 |
| U75390 | putrescine N-methyltransferase [Solanumdulcamara] | 0.00342 | 7.496734 |
| **minor CHO metabolism** | | | |
| U61390 | trehalose-6-phosphate synthase [Ginkgo biloba] | 0.02312 | 7.210233 |
| U69254 | myo-inositol oxygenase [Eucalyptus grandis] | 6.11E-11 | 8.840841 |
| **Mitochondria electron transport** | | | |
| U56488 | cytochrome oxidase subunit I [Salix nigra] | 0.00108 | -7.84367 |
| U54716 | cytochrome oxidase subunit I [Pseudotsugamacrocarpa] | 0.00051 | -8.20212 |
| U58883 | cytochrome c oxidase subunit 1 [Notheiaanomala] | 5.82E-07 | -8.3087 |
| U46418 | cytochrome c oxidase subunit 2 [Beta vulgaris subsp. vulgaris] | 1.70E-13 | -8.50851 |
| U57426 | cytochrome c oxidase subunit 1 [Pythiumultimum] | 6.67E-10 | -9.35136 |
| U54656 | NADH dehydrogenase subunit 1 [Cyanidioschyzonmerolae] | 4.51E-18 | -9.4571 |
| **Nucleotide metabolism** | | | |
| U56754 | Thymidylate kinase OS=Bradyrhizobiumjaponicum GN=tmk PE=3 SV=1 | 0.01031 | -7.21965 |
