# Supplementary figures and images for: In-Depth Transcriptome Sequencing of Mexican Lime Trees Infected with Candidatus Phytoplasma aurantifolia
Source: PLoS One. 2015 Jul 1;10(7):e0130425. doi: 10.1371/journal.pone.0130425 (PMC4489016; doi:10.1371/journal.pone.0130425)

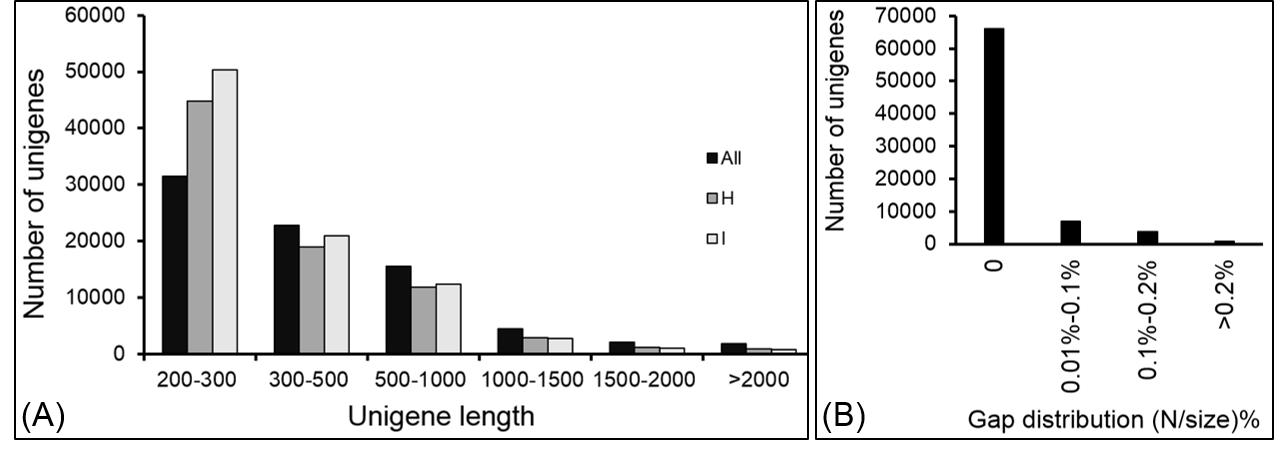


**Figure A**


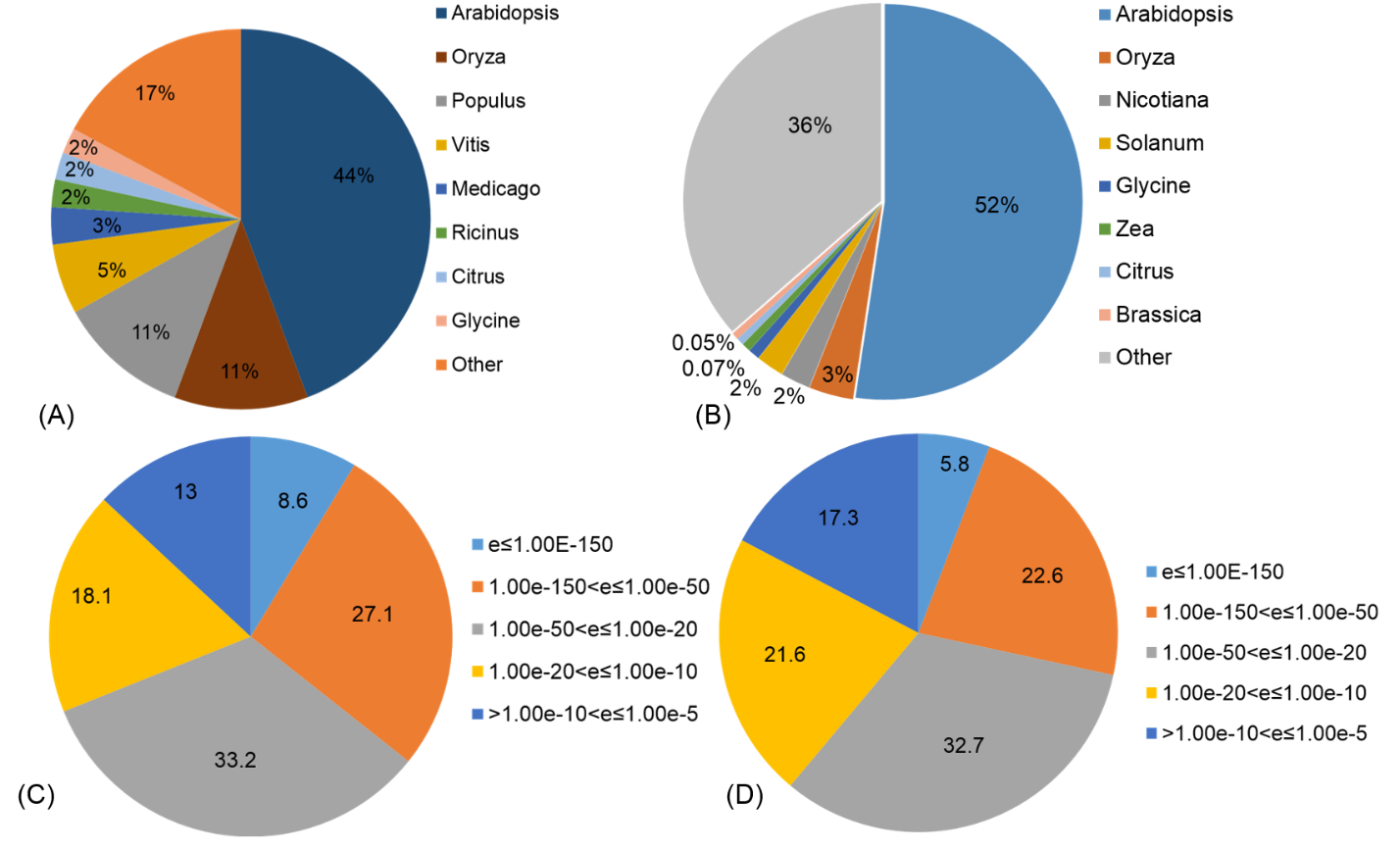


**Figure B**

**
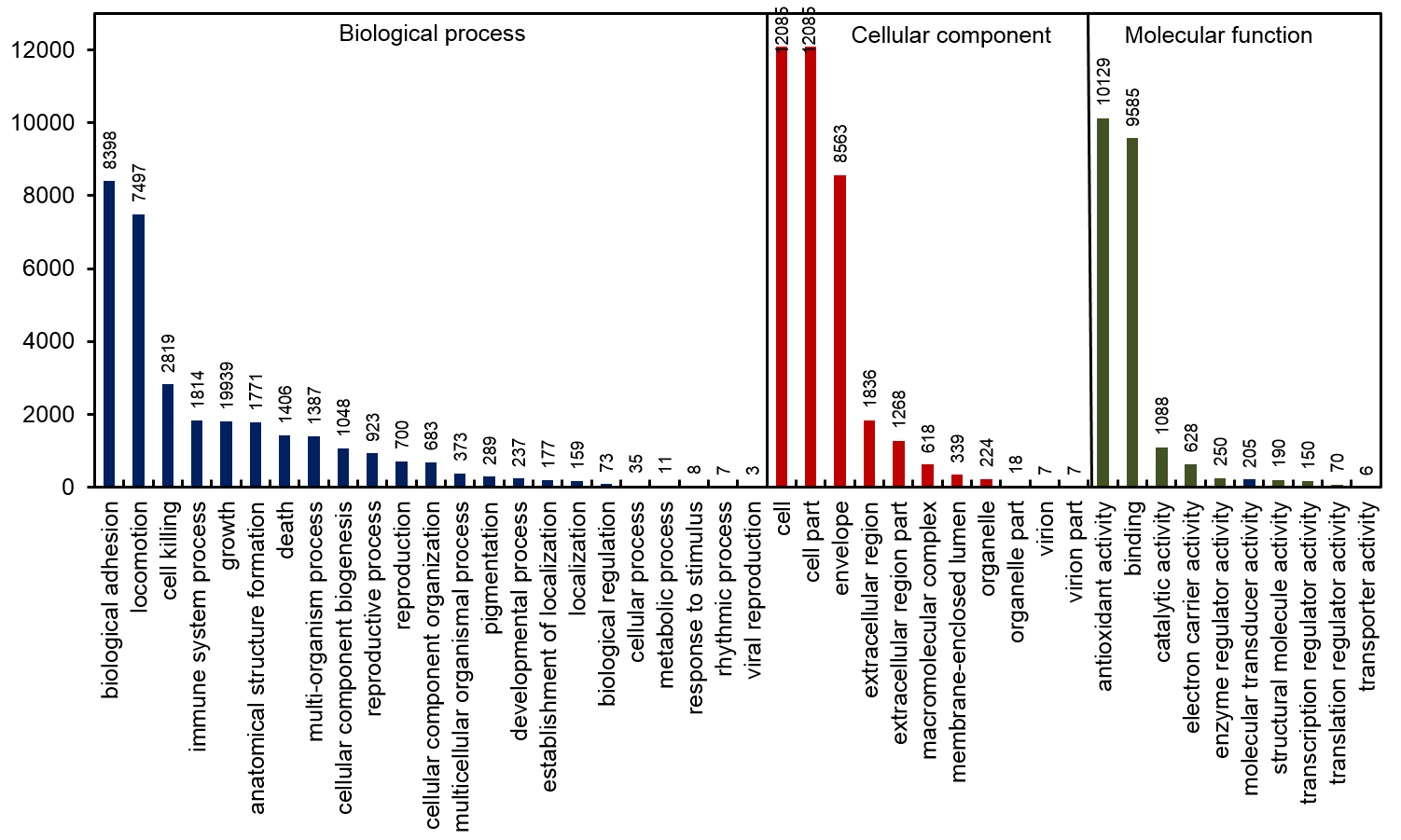
**

**Figure C**


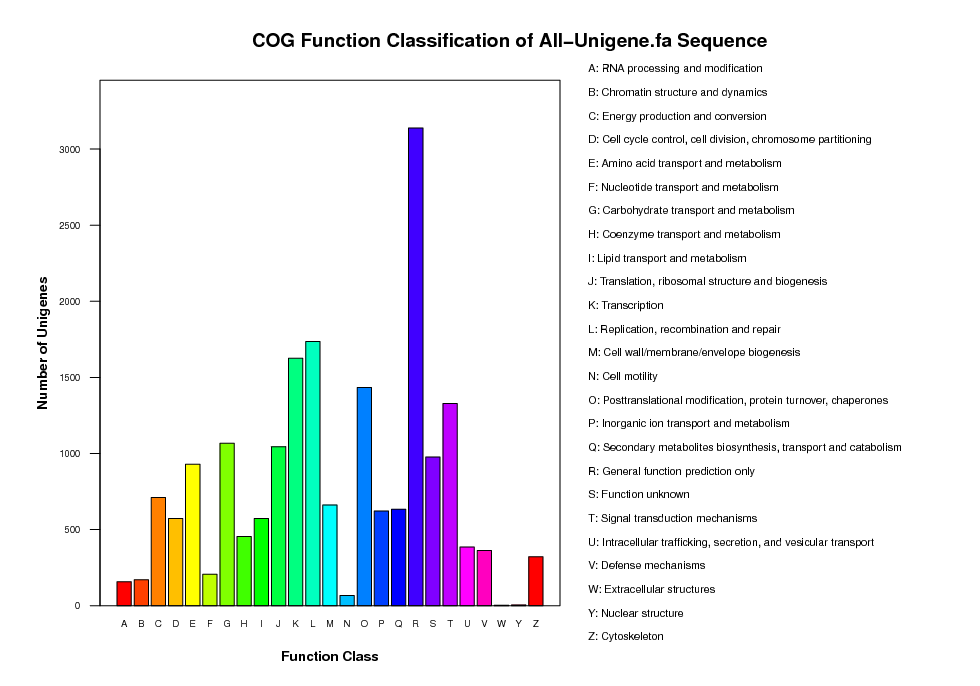


**Figure D**

**
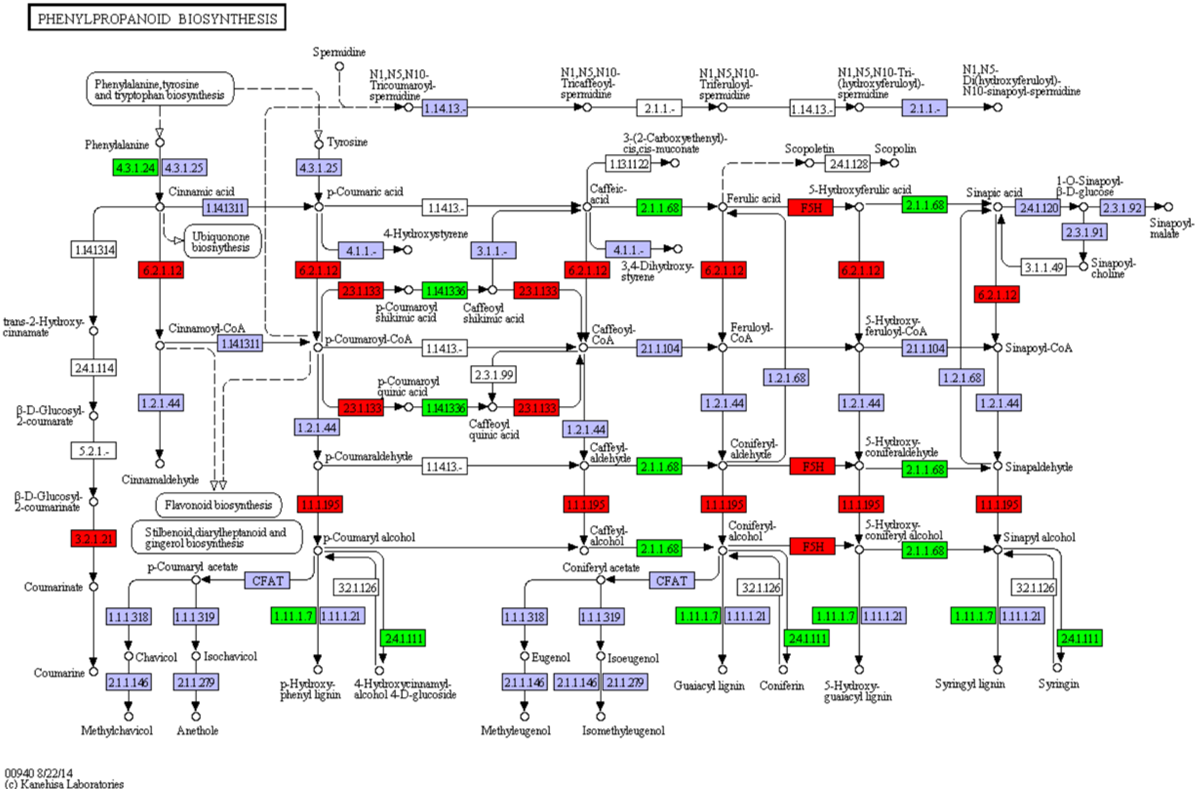
**

**Figure E**

Supplement: S2 File — Figure A in S2 File: (A) Distribution of the length of unigenes in infected (I), healthy (H), and pooled libraries (All). A significant proportion of unigenes were between 200 and 300 nt in length, with most of them remaining unannotated. (B) The number of gaps in unigenes. The gap distribution represents the number of N divided by the sequence length of the assembled unigene. Figure B in S2 File: Species (A and B) and e-value (C and D) distribution of top blast hits from the NCBI non-redundant protein (nr) and Swiss-Prot databases, respectively The majority of unigene were matched with significantly low e-values to sequences from species with well-annotated genomes. Figure C in S2 File: GO functional classification. A total of 18,336 unigenes (23% of total unigenes) were assigned to at least one GO term and classified into the three high-level GO terms: biological processes, cellular components, and molecular functions. Figure D in S2 File: Classification of unigenes based on Cluster of Orthologous Group (COG) of genes. A total of 11,736 unigenes that produced significant hits when probed against the NCBI nr database were classified into 25 functional categories. Figure E in S2 File: KEGG pathway visualisation of differentially expressed unigenes related to the phenylpropanoid biosynthesis pathway. Up-regulated enzymes are coloured green and down-regulated enzymes are coloured red. The majority of enzymes (peroxidase, coniferyl-alcohol glucosyltransferase, and caffeic acid 3-O-methyltransferase) that catalyse the final steps of the biosynthesis of lignin precursors (syringin, syringyl lignin, guaiacyl lignin, 5-hydroxy guaiacyl lignin) were up-regulated in diseased plants. (DOCX) [file pone.0130425.s002.docx]
